# The immune system as a guardian of health: micronutrient support and lifelong protection

**DOI:** 10.3389/fnut.2026.1802025

**Published:** 2026-04-09

**Authors:** Konstantinos Mantantzis, Adeline Pierre, Philip C. Calder

**Affiliations:** 1 Bayer Consumer Care AG, Basel, Switzerland; 2 Faculty of Medicine, School of Human Development and Health, University of Southampton, Southampton, United Kingdom; 3 NIHR Southampton Biomedical Research Center, University Hospital Southampton NHS Foundation Trust and University of Southampton, Southampton, United Kingdom

**Keywords:** immune protection, immune system, life span, micronutrients, minerals, vitamins

## Abstract

The immune system constitutes a physiological network that preserves organismal stability through continuous interactions with tissues and regulatory systems. Immune activity is an integral component of normal tissue physiology, contributing to host defense, structural integrity, metabolic balance, and functional homeostasis. The immune system integrates molecular recognition, effector mechanisms, and inflammation resolution to control infections, limit tissue damage, and reduce the progression of chronic and degenerative diseases. These combined defensive and physiological functions underpin the concept of the immune system as a “guardian of health.” Immune competence varies across the life course, following a dynamic trajectory shaped by development, maturation, stabilization, and age-related decline (immunosenescence). Early life, adolescence, pregnancy, menopause, and aging are characterized by increased immune plasticity and shifting functional demands. In later life, immunosenescence and chronic low-grade inflammation (inflammaging) increase immune vulnerability, to the extent that immune resilience determines interindividual variability with aging and subsequent health outcomes. Micronutrients are central determinants of immune resilience, as they support immune cell proliferation and function, redox balance, epithelial barrier integrity, and inflammation control. Vitamins A, C, D, E, and the B-group, together with trace elements such as zinc, selenium, iron, and copper, act within interconnected metabolic and signaling networks that sustain innate and adaptive immune responses. Despite their essential role, subclinical micronutrient insufficiencies remain common, including in high-income countries, and are associated with reduced immunocompetence and increased susceptibility to infection and inflammation. Overall, this evidence highlights that life-course-based immunonutrition could be a strong strategy to preserve health-span and promote long-term physiological resilience.

## Introduction

The immune system is a physiological network that maintains the functional stability of the host organism. It integrates mechanisms of host defense, molecular recognition, effector responses, and inflammation resolution that limit infection, contain tissue damage, and reduce the progression of chronic and degenerative diseases (1). Taken together, this functional and physiological repertoire supports a view of the immune system that encompasses both defense and repair functions, as well as having an active role in tissue maintenance and overall organismal health. This functional repertoire establishes the immune system as the body's principal biological defense system, supporting its conceptual consideration as a “guardian of health” (2).

Immune integrity is essential to sustain homeostasis in the face of external and internal stimuli. Variations in the functioning of the immune system throughout the life course influence susceptibility to infections, inflammatory dynamics, and age-related changes in health (3). In this context, immune resilience, understood as the capacity of the immune system to maintain effective function under challenge or stress, is associated with an efficient response to infections and age-related stressors (4).

Immune capacity varies significantly across the life span, accompanying the processes of development, maturation, stabilization, and age-related decline (immunosenescence) (3). This dynamic behavior is shaped by the interaction between physiology, environmental exposure, and nutrient availability, the latter being a key determinant of immune activity throughout the life course (5).

The recognition of immunity as a “guardian of health” becomes particularly relevant in aging, a stage in which its function is crucial for preserving health span, defined as healthy life expectancy (6, 7). In this context, immune resilience is a key component of functional maintenance and is shaped by genetic and environmental factors, by immunobiography (infectious and vaccination history), by exposure to medications, and by lifestyle elements such as nutrition and physical activity (8). The integration of these determinants helps strengthen defensive function and reduce susceptibility to infections, autoimmunity, and age-related non-communicable diseases (7).

In parallel with changes in immune capacity, immune requirements vary throughout the life course, and certain stages may be particularly sensitive to nutrient deficiencies. Within this framework, micronutrients contribute to the maintenance of essential immune functions and support the body's capacity to respond to the challenges characteristic of each stage. Despite increasing recognition of the role of micronutrients in supporting immune function, a comprehensive life-course perspective addressing immune resilience from early life to aging is still lacking (9). The present work aims to examine how immunity safeguards health across different phases of life and how micronutrients contribute to maintaining optimal immune functioning (10).

### The immune system as a guardian of health

Immune activity constitutes an integral component of tissue physiology, exerting continuous regulatory control over normal organ function. Through coordinated interactions with parenchymal and stromal compartments, resident immune cells (particularly innate populations such as macrophages) contribute to the maintenance of tissue structure and functional integrity. These homeostatic immune processes regulate fundamental physiological functions, including bone remodeling, muscle performance and metabolic homeostasis, thereby sustaining normal tissue physiology in the absence of infection or inflammatory challenge (11, 12).

**Figure 1:**
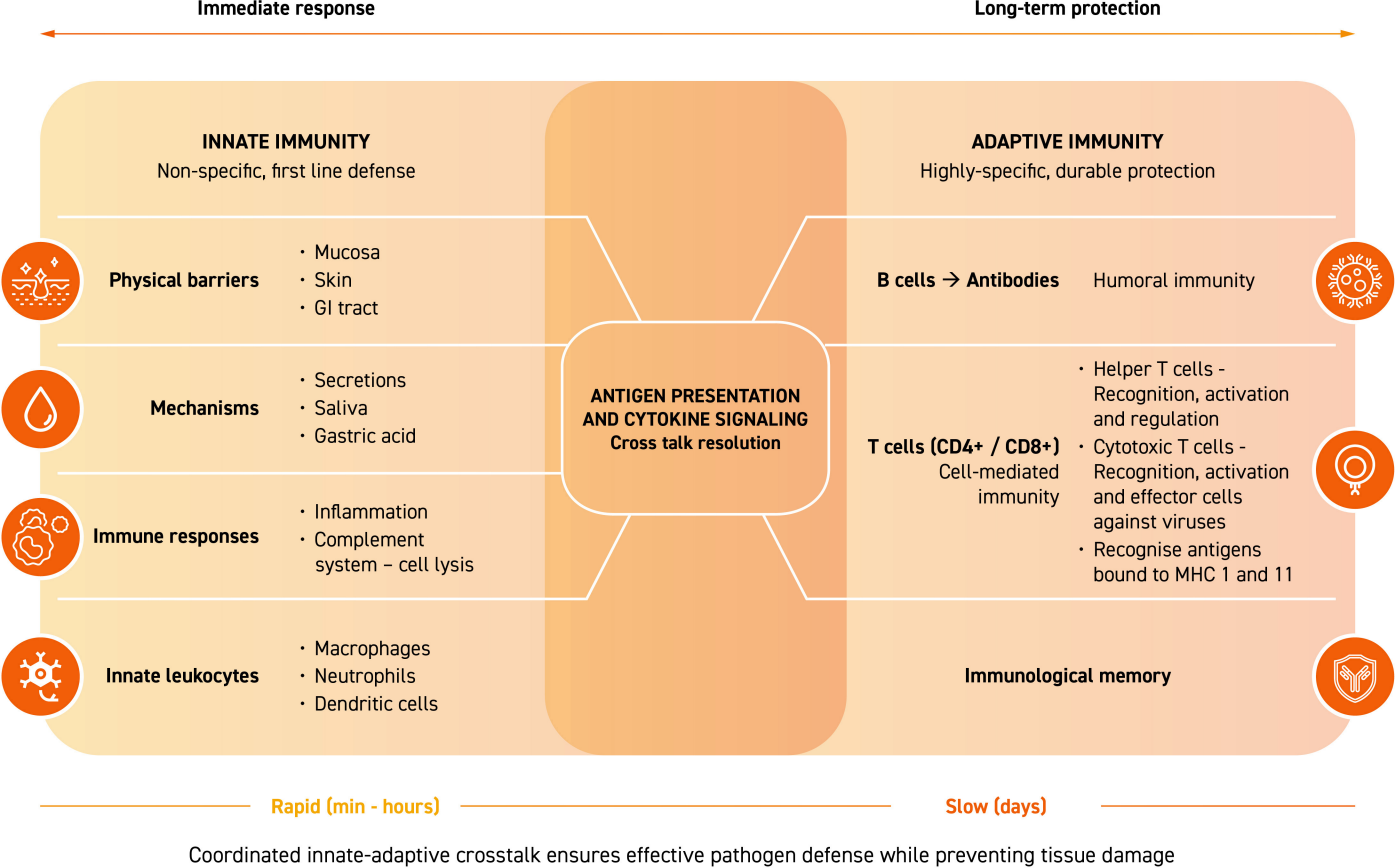
Integrated and bidirectional organization of host defense by the immune system.

At a functional level, host defense is organized as an integrated network that combines the rapidity of innate immunity with the specificity of adaptive immunity (Figure 1). Specifically, innate immunity functions as the first line of defense through physical and chemical barriers, pattern-recognition receptors that detect conserved pathogen structures, and effector cell populations (including phagocytic cells) (13). In parallel, adaptive immunity deploys specific responses mediated by T and B cells, whose activation leads to the generation of antibodies, immunological memory and more durable protection against possible future exposure to pathogens. Bidirectional communication between innate and adaptive immune compartments is essential to orchestrate an appropriate immune response: signals derived from innate activation shape the magnitude of adaptive responses, while activated T lymphocytes modulate the function of innate components to fine-tune the response and limit damage to healthy cells and tissues. This dynamic balance allows for efficient defense against pathogens without triggering dysregulated inflammatory processes that compromise tissue integrity (14).

The immune system coordinates, in an integrated manner, defense against pathogens, control of inflammation, and tissue repair processes. Following infectious or physical injury, the initial inflammatory response aims to eliminate the damaging agent and generates signals that activate reparative cells (15). Macrophages act as central regulators of this transition due to their plasticity: after adopting pro-inflammatory phenotypes required for early clearance, they evolve into pro-resolutive profiles that promote angiogenesis, extracellular matrix remodeling, and the recovery of tissue function (16). This succession of events ensures effective resolution of damage and prevents pathological outcomes such as fibrosis or chronic inflammation, demonstrating that immune defense mechanisms and repair programs are interdependent processes (17).

Physiological defense requires the activation of effective defensive mechanisms without inducing pathological inflammation. This balance depends on highly integrated cellular and molecular regulatory networks that modulate the intensity, duration, and resolution of the immune response (18). The immune system functions as an interdependent network in which the integration of signals from different cell types and molecular mediators adjusts the magnitude of the response proportionally to the stimulus. Disruption of these processes can lead to allergy, autoimmunity, chronic inflammation, or tissue degeneration, highlighting the importance of maintaining a stable immune equilibrium to preserve homeostasis (11).

Defense mechanisms are tightly interconnected with other physiological systems that continuously modulate immune activity. The intestinal microbiome influences the maturation and regulation of the immune system through bioactive metabolites and signals derived from the mucosal epithelium (12). In parallel, hormones of the endocrine system adjust the thresholds for inflammatory activation and regulate the functionality of multiple lymphocyte populations (19). Likewise, the nervous system participates through neuroimmune circuits that integrate stimuli related to stress, emotional state, and defensive responses (20). Collectively, these interactions show that immunity is a dynamic component of systemic physiology and that immune activity depends on the integration of signals originating from different organs (11, 12, 18).

### Immune function across the life span

In the earliest stages of human development, immune function is strongly influenced by maternal factors, including the placental transfer of IgG and the nutritional and immunological contributions of breastfeeding (3, 21). After birth, breast milk provides secretory IgA, antimicrobial peptides, and immunoregulatory mediators that reinforce mucosal barriers, shape early interactions between the microbiota and epithelial tissues and promote immune maturation (22–24). Together with early microbial exposure and vaccination, these inputs act during a period of high immune plasticity to establish the foundations of immune tolerance and adaptive competence, which are subsequently refined during later early-life developmental stages (3, 22).

During childhood, the immune system acquires greater functional stability. Mucosal barriers undergo structural consolidation, strengthening local defense and reducing the need for systemic inflammatory responses. Acquired immunity increases in precision through higher-affinity antibodies, expansion of memory cells, and more coordinated effector responses (22). These changes reduce the inflammation associated with common infections and increase resilience to environmental stimuli. Sustained interaction between the microbiota, nutrition, and behavior maintains the balance between tolerance and activation, consolidating the regulatory mechanisms established in early life (21).

In environments characterized by substantial pathogen exposure, such as highly polluted settings, recurrent immune activation entails considerable energetic costs. Lymphocyte proliferation, synthesis of immune mediators, and fever require metabolic resources that directly compete with those needed for somatic growth, particularly linear growth (25). This trade-off prioritizes immediate immune defense over investment in growth and may result in reduced stature or altered developmental trajectories. Competition between immune function and other physiological systems for limited resources is a key determinant of growth patterns and long-term health outcomes (26). In real-world contexts, these growth-related trade-offs often coexist with additional environmental stressors, including air pollution, which has been independently associated with impairments in cognitive functions such as attention, memory, processing speed, and psychomotor performance in children and adolescents (27).

Adolescence introduces a second critical phase of immune reorganization. Hormonal changes associated with puberty modify immune reactivity and generate sex differences in inflammatory regulation and susceptibility to infections (28). Pubertal growth involves sex-specific body changes, including increased body mass in males and the establishment of the menstrual cycle in females, which alter the hormonal and metabolic environment in which the immune system operates (29). Adolescence is also accompanied by neuroimmune adjustments and heightened sensitivity to stress, nutrition, and social context, factors that exert persistent effects on immune regulation (30).

During adulthood, the immune system reaches a period of relative stability in which the maintenance of immunocompetence is strongly influenced by environmental factors, metabolic status, and lifestyle. Evidence indicates that exposure to air pollutants, sustained work-related demands, psychosocial stress, and disruptions in circadian rhythms contribute to a gradual increase in baseline inflammation and compromise immune resilience, reducing the capacity to recover after an immune challenge (31). Metabolic determinants add to this burden: weight control, adipose tissue distribution, insulin sensitivity, and gut microbiota composition play central roles in regulating immune function (32, 33). Immune homeostasis depends on the body's ability to buffer the impact of chronic exposures that, over time, can alter the system's functional equilibrium. In this context, the capacity of the immune system to continually modulate and compensate for the impact of these chronic exposures decisively shapes subsequent immune trajectories (34).

Within adulthood, pregnancy represents a distinct physiological state characterized by a finely regulated recalibration of immune function (35). During gestation, a tolerogenic immune profile supports implantation and fetal development while maintaining antimicrobial defense through controlled modulation of inflammatory responses, accompanied by metabolic and hormonal changes that influence immune cell distribution and baseline inflammation (36).

After the reproductive stage, the female menopausal period marks a significant shift in the intersection between the endocrine system, metabolism and immunity. The decline in levels of estrogen, which modulates immune responses and inflammatory control, is associated with an increase in baseline inflammatory tone, changes in body composition, and worsened insulin sensitivity (37). In parallel, alterations in gut microbiota and in the communication between adipose tissue and the immune system have been described to occur during menopause. In women, menopause is recognized as an inflection point in the trajectory toward immune aging (38).

In old age, the immune system enters a phase dominated by two closely interrelated processes: immunosenescence and inflammaging (39–42). Over time, the capacity of the immune system to generate new responses declines, while low-grade inflammation remains chronically elevated. This dual phenomenon affects responses to infections (43, 44) and vaccines (45–47) and alters the way the immune system interacts with other tissues. In the brain, the inflammatory environment contributes to progressive cognitive decline and less efficient regulation of neuronal energy metabolism (48). Persistent inflammatory signaling also affects the cardiovascular system, contributing to endothelial dysfunction and promoting processes associated with vascular aging, such as increased arterial stiffness (49, 50). At a metabolic level, maintaining glycemic control becomes more challenging, with increased insulin resistance (51). These mechanisms also influence musculoskeletal tissues: muscle regeneration becomes less effective and bone remodeling becomes imbalanced, increasing loss of muscle mass and bone fragility, with rising risks of sarcopenia and osteoporosis (52, 53). While immunosenescence and inflammaging are often detrimental, they are also accompanied by adaptive and homeostatic adjustments that shape the variability of immune aging across individuals, such as enhanced immune resilience, compensatory activation of innate defense pathways, and microbiota-mediated modulation of low-grade inflammation, which can help preserve tissue function and immune balance in later life (6, 54, 55).

### Factors influencing immune competence

Immune competence is the result of dynamic interactions among life factors such as nutritional status, psychological stress, physical fitness and microbiome composition alongside genetic and epigenetic influences, circadian rhythms, and history of microbial exposure. Both undernutrition and over nutrition alter lymphocyte proliferation, phagocytic function, and cytokine profiles, thereby compromising the effectiveness of both innate and adaptive immune responses (56). Sleep deprivation reduces natural killer cell activity and promotes a pro-inflammatory state (57). Sustained stress persistently activates the hypothalamic–pituitary–adrenal axis, increasing release of glucocorticoids, which suppress adaptive immunity (58). Although exercise and being physically fit enhance immunosurveillance, excessive physical loads can induce transient immunosuppression (59). In this context, adverse alterations in the gut microbiota further aggravate immune dysfunction by impairing proper immune maturation, destabilizing tolerance mechanisms, and reducing the production of essential immunomodulatory metabolites, thereby promoting a chronic inflammatory state (60).

Lifestyle and environmental factors exert a substantial influence on immune homeostasis. Continuous exposure to atmospheric pollutants and environmental toxins characteristic of dense urban settings promotes oxidative stress, disruption of epithelial barriers, and alterations in the expression of genes encoding immune mediators with downstream consequences for immune regulation and overall health across the lifespan (61). In parallel, habits such as smoking and excessive alcohol consumption foster a chronic inflammatory state and compromise key mechanisms of immunosurveillance (62). Differences in environmental exposures and behavioral factors modulate interindividual variability in immune responses, shaping susceptibility to infections, autoimmunity, and inflammatory diseases (63).

Chronic diseases and metabolic alterations are key determinants of immunocompetence. Obesity, insulin resistance, and type 2 diabetes generate persistent systemic inflammation driven by adipokines, oxidative stress, and metabolic dysfunction (64). These processes reduce T cell diversity and functionality, alter antigen presentation, and weaken vaccine responses (65). In addition, metabolic dysregulation accelerates immunosenescence, promoting a chronic inflammatory phenotype and increasing susceptibility to infections (66–69). Caloric restriction and metabolic improvement have been shown to restore immune functions and decrease inflammatory markers, reinforcing the close relationship between metabolism and immunity (70).

Among all modifiable lifestyle determinants, nutrition, particularly micronutrient intake and status, constitutes a central and integrative axis for immune competence. Micronutrients are closely linked to immune outcomes and to multiple interconnected risk factors that shape immune homeostasis, including metabolic health and inflammatory burden (5, 9, 71). By modulating these shared pathways, adequate nutritional status may generate a positive cascading effect that supports immune function across multiple complementary biological systems. Chronic inflammation and oxidative stress increase the metabolic consumption of key vitamins and minerals, depleting stores, and reducing their availability for supporting immune functions. Alterations in the gut microbiota can impair micronutrient absorption and the synthesis of metabolites with immunoregulatory roles (56). Vitamins A, C, B6, B9, B12, D, and E, together with trace elements such as zinc, selenium, iron, and copper, are essential for lymphocyte maturation and functionality, the maintenance of epithelial integrity, and the regulation of redox balance (5, 9, 56, 72). When the equilibrium of these micronutrients is disrupted by increased oxidative and inflammatory stress and by the heightened metabolic demand associated with these stresses, a state of greater immune vulnerability emerges, increasing the need for targeted micronutrient support to restore homeostasis and sustain immune resilience (72).

### Micronutrients and immune support

Micronutrients are essential determinants of immune competence because they participate in metabolic pathways and signaling systems that shape the effectiveness of immune responses (5, 9, 71, 73). Among vitamins, B-group vitamins (B6, B9, B12) support essential metabolic processes required for the rapid proliferation of immune cells during an immune response, thereby sustaining effective lymphocyte expansion following activation (74, 75). Vitamin E supports immune competence by limiting oxidative damage and sustaining T cell–mediated responses during immune activation (5, 72). Vitamin D plays a key role in immune regulation by shaping antigen presentation and enhancing innate antimicrobial defenses, contributing to protection against infections, particularly those affecting the respiratory tract (76–80). Vitamin C supports antioxidant defense during immune activation and helps preserve epithelial barrier integrity, which is critical for preventing pathogen entry (79, 81). Vitamin A, through its active metabolites, contributes to the maintenance of epithelial surfaces, mucosal immunity, IgA production, and the balanced differentiation of T and B cells (82).

**Figure 2:**
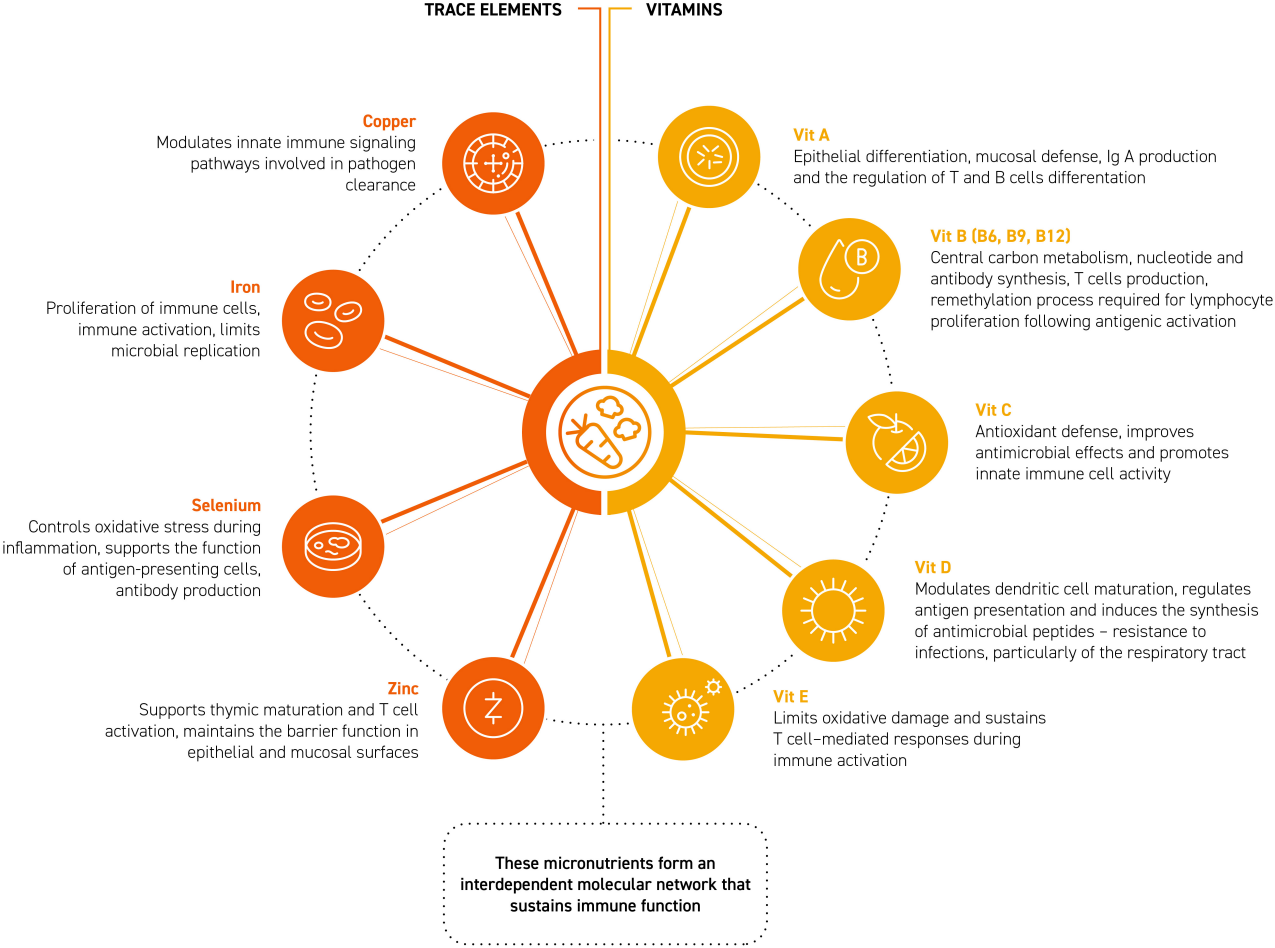
Role of micronutrients in supporting immune functions.

Among trace elements, zinc acts as a cofactor that supports thymic maturation and T cell activation, while also promoting the maintenance of barrier function in epithelial and mucosal surfaces (83, 84). Zinc also seems to have some specific anti-viral actions (85). Selenium, incorporated into antioxidant selenoproteins, contributes to the control of oxidative stress during inflammation and supports the function of antigen-presenting cells and T cells (86) and, again may have anti-viral actions (87, 88). Iron, which is indispensable for energy metabolism and the proliferation of immune cells, participates in redox reactions essential for immune activation, whose tight regulation also limits microbial replication (89). Similarly, copper participates in redox reactions and in the activity of antimicrobial oxidases, modulating innate immune signaling pathways involved in pathogen clearance (90). Collectively, these micronutrients form an interdependent molecular network that sustains immune function (5, 9, 71, 73) (Figure 2).

Recent biomarker-based data indicate that insufficiency of essential micronutrients (such as vitamins A, B6, B9, and B12, C, and the trace elements zinc, selenium, iron, and copper) remains common, even in high-income countries (91–94). The literature shows that deficiencies in these micronutrients are associated with poorer immune resilience (9, 73, 94–96). Furthermore, clinical evidence reveals a consistent pattern: when subclinical insufficiency is present, correcting vitamin and mineral status through supplementation increases serum concentrations of those micronutrients and restores immune parameters (56, 97–99). The combination of vitamin C and zinc has been shown to reduce the duration and severity of the common cold and to induce transcriptomic changes compatible with a more efficient inflammatory response (100–105). These findings align with studies evaluating multivitamin-mineral complexes, in which correcting insufficient status for vitamin C, zinc, selenium, and B vitamins is associated with enhanced natural killer cells activity, improved antibody responses, and reduced incidence of infections (5, 9, 71, 97, 106).

Micronutrient requirements are higher in specific subgroups, particularly in individuals with metabolic syndrome, who frequently exhibit lower plasma concentrations of vitamins A, B6, B9, B12, and C, and the trace elements zinc and selenium (94, 96, 107). Among these, vitamin C provides a clear illustrative example, as metabolic syndrome is associated with increased vitamin C requirements and a reduced likelihood of achieving adequate circulating levels through habitual intake (108). Insufficient vitamin C status in this population contributes to heightened oxidative stress and sustained low-grade inflammation, thereby exacerbating immune vulnerability and metabolic dysfunction (108). Consistent with this, evidence indicates that a substantial proportion of individuals with metabolic syndrome fail to meet recommended micronutrient intakes, reinforcing the relevance of targeted nutritional strategies in this high-risk group (94, 96, 107, 108). This increased vulnerability accelerates micronutrient utilization, contributing to an abrupt decrease in concentrations, especially during infections when metabolic demand rises. Supplementation is essential to restore serum levels and sustain immune responses (5, 71, 107, 108).

In this setting, “adequate intake” refers to achieving current dietary reference intakes [e.g., recommended dietary allowances (RDAs)], which are established to prevent deficiency; however, evidence indicates that for optimal immune function, requirements for key micronutrients (vitamins A, C, D, E, B6, B9, and B12, and the trace elements zinc, selenium, iron, and copper) may exceed these values, particularly during periods of physiological stress or infection (109). Quantitative data are available for several of these micronutrients, indicating higher intakes than the RDA, including vitamin C at ≥200 mg/day to ensure immune cell saturation (81), vitamin D at intakes often ≥1,000–2,000 IU/day to achieve serum levels associated with reduced infection risk, zinc at 15–30 mg/day in the context of immune support, vitamin E at approximately 200 mg/day to enhance T cell function in older adults (110), and selenium at around 100–200 μg/day to optimize selenoprotein activity (111), supporting the concept that micronutrient requirements for immune function may be substantially higher than those required to prevent deficiency.

**Figure 3:**
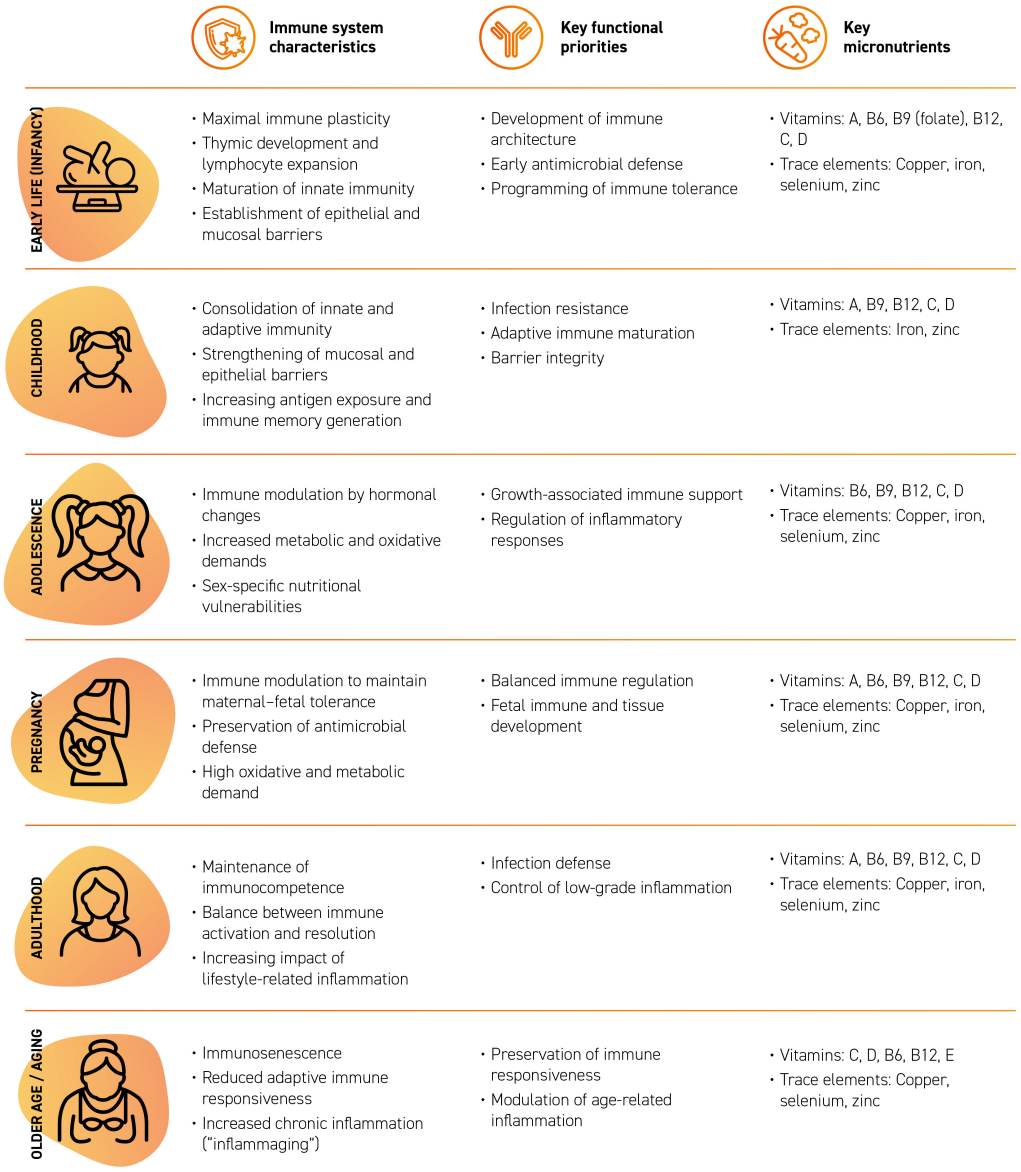
Characteristics of the immune system across the life span, key functions and key micronutrients.

The micronutrient intakes needed to sustain optimal immune function vary throughout the life course (112) (Figure 3). The earliest stages of development represent a period of maximal immune plasticity, during which thymic formation, lymphocyte expansion, and the maturation of innate immunity depend on adequate supplies of vitamins A, C, D, and B-group vitamins, as well as zinc, iron, selenium, and copper (113). In childhood, the consolidation of mucosal barriers and the maturation of adaptive immunity require sufficient intakes of vitamins A, C, D, B9, and B12, together with minerals such as zinc and iron (114).

During adolescence, hormonal changes influence immune function: in males, growth, muscle development, and increased metabolic demands make adequate intake of vitamin D and B-group vitamins, along with zinc, iron, selenium, and copper, particularly relevant; in females, menstruation increases the importance of iron and appropriate intake of vitamins B9 and B12, together with vitamin D, zinc, and selenium (114, 115).

During pregnancy, the immune modulation required for maternal–fetal tolerance increases the need for vitamins A, C, D, and B-group vitamins, together with zinc, iron, selenium, and copper, which are essential for fetal development, inflammatory balance and the maintenance of maternal immune responses (115, 116).

In adulthood, maintaining immunocompetence depends on an adequate intake of vitamins A, C, and D, B-group vitamins, and minerals such as zinc, selenium, iron, and copper (112, 115). After menopause in women and with aging, immunosenescence increases the importance of vitamins C, D and E, B-group vitamins, and zinc, selenium, and copper, which are necessary to preserve immune function and moderate age-related inflammation (71, 117).

### Clinical and public health implications

Evidence suggests that the dietary reference intakes (e.g., RDAs) for micronutrients do not reflect the levels required to maintain optimal immune resilience, as they were designed to prevent deficiencies rather than to maximize immune functionality. Clinical studies indicate that micronutrients such as vitamins C and D, zinc, and selenium are required in concentrations higher than those proposed in classical guidelines to sustain mucosal barrier integrity, modulate cytokine production, and optimize immunosurveillance (9). Further to this, individuals who meet the RDA are still observed to experience recurrent infections (9, 118). Translating these findings into clinical recommendations requires considering targeted supplementation and reassessing official thresholds, especially in populations with greater physiological demands or associated risks (118).

Maintaining immune resilience requires multimodal preventive strategies that prioritize adequate micronutrient intake, particularly during periods of increased vulnerability such as childhood, pregnancy, menopause, aging, or the presence of comorbidities (119). Evidence indicates that key micronutrients, including vitamins C and D and zinc, act in complementary and synergistic ways to support antiviral defenses, maintain epithelial barrier function, and modulate inflammatory responses. Together, these micronutrient interactions contribute to greater immune efficiency and help limit low-grade systemic inflammation, reinforcing immune resilience across the life course (120).

Vaccination represents a cornerstone of preventive healthcare, providing protection against infectious diseases by priming adaptive immune responses and generating immunological memory (121). However, vaccine efficacy can be influenced by host-related factors, including age, comorbidities, and nutritional status. In particular, older adults and individuals with compromised immune function may exhibit reduced vaccine responses, partly due to immunosenescence and underlying micronutrient deficiencies (122).

A growing body of evidence indicates that inadequate micronutrient status is associated with impaired responses to vaccination, whereas ensuring adequate nutritional status has been reported to enhance vaccine immunogenicity and effectiveness, particularly in vulnerable populations. Combining vaccination strategies with adequate nutrition may represent a practical and cost-effective approach to optimize immune protection and improve public health outcomes (122).

The integration of these findings into public health policies is essential for the prevention of chronic diseases associated with persistent inflammation and immune deterioration. Immunonutrition incorporated into population programs would improve the functional capacity of the immune system and contribute to healthy aging through sustainable and cost-effective interventions (95, 123).

In this regard, healthcare professionals are key agents in translating theoretical evidence into clinical application. Their involvement in nutritional education, screening for deficiencies and personalized guidance is fundamental to improving adherence, counteracting misinformation and promoting immune self-care (10, 124).

## Potential limitations

Certain limitations should be acknowledged. The available evidence in this field is heterogeneous and continues to evolve, which may influence the interpretation of current findings. Further research is warranted to strengthen the evidence base and refine current understanding of the role of micronutrients in immune function across the life course.

## Conclusions

The immune system plays a central role as a guarantor of host defense, physiological stability, preservation of tissue integrity and organ function, modulation of inflammation and protection against pathogens across the lifespan. This foundational role underpins its designation as the primary guardian of health.

Based on the mechanisms described, current evidence indicates that nutritional sufficiency, including adequate micronutrient support, and healthy lifestyle patterns are critical determinants of immune resilience in the context of aging, as well as metabolic and environmental challenges. Importantly, the persistence of subclinical vitamin and mineral insufficiencies across different age groups, including in high-income countries with broad food availability, indicates that immune vulnerability can develop silently. This hidden impairment contributes to increased susceptibility to infections and chronic diseases, with downstream consequences for healthcare systems, including higher disease burden, increased healthcare utilization, and rising long-term economic costs (94).

Collectively, these findings highlight that adequate micronutrient status is essential to support immune resilience across the life course and to reduce susceptibility to infection and disease. These findings have important implications for research, policy, and clinical practice, supporting the need to integrate a life-course–based immune health framework.

These considerations support the need to integrate a life-course–based immune health framework across research, policy, and clinical practice. In practical terms, this involves incorporating immune-supportive nutrition and micronutrient adequacy into public health strategies, preventive care, and clinical guidelines, while prioritizing research that informs evidence-based thresholds and targeted interventions. Such an end-to-end approach has the potential to strengthen immune adaptability, reduce preventable disease burden, and support sustainable healthy aging at both individual and population levels.
